# Predicting Short-term MCI-to-AD Progression Using Imaging, CSF, Genetic Factors, Cognitive Resilience, and Demographics

**DOI:** 10.1038/s41598-019-38793-3

**Published:** 2019-02-19

**Authors:** Yogatheesan Varatharajah, Vijay K. Ramanan, Ravishankar Iyer, Prashanthi Vemuri, For the Alzheimer’s Disease Neuroimaging Initiative, Michael W. Weiner, Paul Aisen, Ronald Petersen, Clifford R. Jack, Andrew J. Saykin, William Jagust, John Q. Trojanowki, Arthur W. Toga, Laurel Beckett, Robert C. Green, John Morris, Leslie M. Shaw, Zaven Khachaturian, Greg Sorensen, Maria Carrillo, Lew Kuller, Marc Raichle, Steven Paul, Peter Davies, Howard Fillit, Franz Hefti, David Holtzman, M. Marcel Mesulam, William Potter, Peter Snyder, Adam Schwartz, Tom Montine, Ronald G. Thomas, Michael Donohue, Sarah Walter, Devon Gessert, Tamie Sather, Gus Jiminez, Archana B. Balasubramanian, Jennifer Mason, Iris Sim, Danielle Harvey, Matthew Bernstein, Nick Fox, Paul Thompson, Norbert Schuff, Charles DeCArli, Bret Borowski, Jeff Gunter, Matt Senjem, David Jones, Kejal Kantarci, Chad Ward, Robert A. Koeppe, Norm Foster, Eric M. Reiman, Kewei Chen, Chet Mathis, Susan Landau, Nigel J. Cairns, Erin Franklin, Lisa Taylor-Reinwald, Virginia Lee, Magdalena Korecka, Michal Figurski, Karen Crawford, Scott Neu, Tatiana M. Foroud, Steven Potkin, Kelley Faber, Sungeun Kim, Kwangsik Nho, Leon Thal, Neil Buckholtz, Marilyn Albert, Richard Frank, John Hsiao, Jeffrey Kaye, Joseph Quinn, Lisa Silbert, Betty Lind, Raina Carter, Sara Dolen, Lon S. Schneider, Sonia Pawluczyk, Mauricio Beccera, Liberty Teodoro, Bryan M. Spann, James Brewer, Helen Vanderswag, Adam Fleisher, Judith L. Heidebrink, Joanne L. Lord, Sara S. Mason, Colleen S. Albers, David Knopman, Kris Johnson, Rachelle S. Doody, Javier Villanueva-Meyer, Valory Pavlik, Victoria Shibley, Munir Chowdhury, Susan Rountree, Mimi Dang, Yaakov Stern, Lawrence S. Honig, Karen L. Bell, Beau Ances, Maria Carroll, Mary L. Creech, Erin Franklin, Mark A. Mintun, Stacy Schneider, Angela Oliver, Daniel Marson, David Geldmacher, Marissa Natelson Love, Randall Griffith, David Clark, John Brockington, Erik Roberson, Hillel Grossman, Effie Mitsis, Raj C. Shah, Leyla deToledo-Morrell, Ranjan Duara, Maria T. Greig-Custo, Warren Barker, Chiadi Onyike, Daniel D’Agostino, Stephanie Kielb, Martin Sadowski, Mohammed O. Sheikh, Anaztasia Ulysse, Mrunalini Gaikwad, P. Murali Doraiswamy, Jeffrey R. Petrella, Salvador Borges-Neto, Terence Z. Wong, Edward Coleman, Steven E. Arnold, Jason H. Karlawish, David A. Wolk, Christopher M. Clark, Charles D. Smith, Greg Jicha, Peter Hardy, Partha Sinha, Elizabeth Oates, Gary Conrad, Oscar L. Lopez, Mary Ann Oakley, Donna M. Simpson, Anton P. Porsteinsson, Bonnie S. Goldstein, Kim Martin, Kelly M. Makino, M. Saleem Ismail, Connie Brand, Adrian Preda, Dana Nguyen, Kyle Womack, Dana Mathews, Mary Quiceno, Allan I. Levey, James J. Lah, Janet S. Cellar, Jeffrey M. Burns, Russell H. Swerdlow, William M. Brooks, Liana Apostolova, Kathleen Tingus, Ellen Woo, Daniel H. S. Silverman, Po H. Lu, George Bartzokis, Neill R Graff-Radford, Francine Parfitt, Kim Poki-Walker, Martin R. Farlow, Ann Marie Hake, Brandy R. Matthews, Jared R. Brosch, Scott Herring, Christopher H. van Dyck, Richard E. Carson, Martha G. MacAvoy, Pradeep Varma, Howard Chertkow, Howard Bergman, Chris Hosein, Sandra Black, Bojana Stefanovic, Curtis Caldwell, Ging-Yuek Robin Hsiung, Benita Mudge, Vesna Sossi, Howard Feldman, Michele Assaly, Elizabeth Finger, Stephen Pasternack, Irina Rachisky, John Rogers, Dick Trost, Andrew Kertesz, Charles Bernick, Donna Munic, Emily Rogalski, Kristine Lipowski, Sandra Weintraub, Borna Bonakdarpour, Diana Kerwin, Chuang-Kuo Wu, Nancy Johnson, Carl Sadowsky, Teresa Villena, Raymond Scott Turner, Kathleen Johnson, Brigid Reynolds, Reisa A. Sperling, Keith A. Johnson, Gad Marshall, Jerome Yesavage, Joy L. Taylor, Barton Lane, Allyson Rosen, Jared Tinklenberg, Marwan N. Sabbagh, Christine M. Belden, Sandra A. Jacobson, Sherye A. Sirrel, Neil Kowall, Ronald Killiany, Andrew E. Budson, Alexander Norbash, Patricia Lynn Johnson, Thomas O. Obisesan, Saba Wolday, Joanne Allard, Alan Lerner, Paula Ogrocki, Curtis Tatsuoka, Parianne Fatica, Evan Fletcher, Pauline Maillard, John Olichney, Charles DeCarli, Owen Carmichael, Smita Kittur, Michael Borrie, T.-Y. Lee, Rob Bartha, Sterling Johnson, Sanjay Asthana, Cynthia M. Carlsson, Pierre Tariot, Anna Burke, Ann Marie Milliken, Nadira Trncic, Adam Fleisher, Stephanie Reeder, Vernice Bates, Horacio Capote, Michelle Rainka, Douglas W. Scharre, Maria Kataki, Brendan Kelly, Earl A. Zimmerman, Dzintra Celmins, Alice D. Brown, Godfrey D. Pearlson, Karen Blank, Karen Anderson, Laura A. Flashman, Marc Seltzer, Mary L. Hynes, Robert B. Santulli, Kaycee M. Sink, Leslie Gordineer, Jeff D. Williamson, Pradeep Garg, Franklin Watkins, Brian R. Ott, Geoffrey Tremont, Lori A. Daiello, Stephen Salloway, Paul Malloy, Stephen Correia, Howard J. Rosen, Bruce L. Miller, David Perry, Jacobo Mintzer, Kenneth Spicer, David Bachman, Nunzio Pomara, Raymundo Hernando, Antero Sarrael, Susan K. Schultz, Karen Ekstam Smith, Hristina Koleva, Ki Won Nam, Hyungsub Shim, Norman Relkin, Gloria Chaing, Michael Lin, Lisa Ravdin, Amanda Smith, Balebail Ashok Raj, Kristin Fargher

**Affiliations:** 1 Department of Electrical and Computer Engineering, University of Illinois at Urbana-Champaign, Urbana, IL 61801 USA; 2 Mayo Clinic, Rochester, MN 55905 USA; 3 University of California, San Francisco, USA; 4 University of Southern California, Los Angeles, USA; 5 Indiana University, Bloomington, USA; 6 University of California, Berkeley, Berkeley, USA; 7 University of Pennsylvania, Philadelphia, USA; 8 University of California, Davis, Davis, USA; 9 Brigham and Women’s Hospital/Harvard Medical School, Boston, USA; 10 Washington University St. Louis, St. Louis, USA; 11 Prevent Alzheimer’s Disease, 2020 Rockville, USA; 12 Siemens, Munich, Germany; 13 Alzheimer’s Association, Illinois, USA; 14 University of Pittsburgh, Pennsylvania, USA; 15 Cornell University, NewYork, USA; 16 Albert Einstein College of Medicine ofYeshiva University, New York, USA; 17 AD Drug Discovery Foundation, New York, USA; 18 Acumen Pharmaceuticals, California, USA; 19 Northwestern University, Illinois, USA; 20 National Institute of Mental Health, Maryland, USA; 21 Brown University, Rhode Island, USA; 22 Eli Lilly, Indiana, USA; 23 University of Washington, Washington, USA; 24 University of California, San Diego, California USA; 25 University of London, London, UK; 26 University of California, Los Angeles, California USA; 27 University of Michigan, Michigan, USA; 28 University of Utah, Utah, USA; 29 Banner Alzheimer’s Institute, Arizona, USA; 30 University of California, Irvine, California, USA; 31 National Institute on Aging, Maryland, USA; 32 Johns Hopkins University, Maryland, USA; 33 Richard Frank Consulting, New Hampshire, USA; 34 Oregon Health and Science University, Oregon, USA; 35 Baylor College of Medicine, Texas, USA; 36 Columbia University Medical Center, New York, USA; 37 University of Alabama-Birmingham, Alabama, USA; 38 Mount Sinai School of Medicine, NewYork, USA; 39 Rush University Medical Center, Rush University, Illinois, USA; 40 Wien Center, Florida, USA; 41 NewYork University, New York, USA; 42 Duke University Medical Center, North Carolina, USA; 43 University of Kentucky, Kentucky, USA; 44 University of Rochester Medical Center, New York, USA; 45 University of Texas Southwestern Medical School, Texas, USA; 46 Emory University, Georgia, USA; 47 University of Kansas, Medical Center, Kansas, USA; 48 Mayo Clinic, Jacksonville, Florida USA; 49 Yale University School of Medicine, Connecticut, USA; 50 McGill University, Montreal- Jewish General Hospital, Quebec, Canada; 51 Sunnybrook Health Sciences, Ontario, Canada; 52 U.B.C. Clinic for AD & Related Disorders, British Columbia, Canada; 53 Cognitive Neurology-St. Joseph’s, Ontario, Canada; 54 Cleveland Clinic Lou Ruvo Center for Brain Health, Ohio, USA; 55 Premiere Research Inst (Palm Beach Neurology), Florida, USA; 56 Georgetown University Medical Center, Washington, D.C USA; 57 Stanford University, California, USA; 58 Banner Sun Health Research Institute, Arizona, USA; 59 Boston University, Massachusetts, USA; 60 Howard University, Washington, D.C USA; 61 Case Western Reserve University, Ohio, USA; 62 Neurological Care of CNY, New York, USA; 63 Parkwood Hospital, Pennsylvania, USA; 64 University of Wisconsin, Wisconsin, USA; 65 Dent Neurologic Institute, New York, USA; 66 Ohio State University, Ohio, USA; 67 Albany Medical College, New York, USA; 68 Hartford Hospital, Olin Neuropsychiatry Research Center, Connecticut, USA; 69 Dartmouth-Hitchcock Medical Center, New Hampshire, USA; 70 Wake Forest University Health Sciences, North Carolina, USA; 71 Rhode Island Hospital, Rhode Island, USA; 72 Butler Hospital, Rhode Island, USA; 73 Medical University South Carolina, Carolina, USA; 74 Nathan Kline Institute, NewYork, USA; 75 University of Iowa College of Medicine, Iowa, USA; 76 USF Health Byrd Alzheimer’s Institute, University of South Florida, Florida, USA

## Abstract

In the Alzheimer’s disease (AD) continuum, the prodromal state of mild cognitive impairment (MCI) precedes AD dementia and identifying MCI individuals at risk of progression is important for clinical management. Our goal was to develop generalizable multivariate models that integrate high-dimensional data (multimodal neuroimaging and cerebrospinal fluid biomarkers, genetic factors, and measures of cognitive resilience) for identification of MCI individuals who progress to AD within 3 years. Our main findings were i) we were able to build generalizable models with clinically relevant accuracy (~93%) for identifying MCI individuals who progress to AD within 3 years; ii) markers of AD pathophysiology (amyloid, tau, neuronal injury) accounted for large shares of the variance in predicting progression; iii) our methodology allowed us to discover that expression of *CR1* (complement receptor 1), an AD susceptibility gene involved in immune pathways, uniquely added independent predictive value. This work highlights the value of optimized machine learning approaches for analyzing multimodal patient information for making predictive assessments.

## Introduction

Alzheimer’s disease (AD) affects approximately 5.5 million people in the United States and more than 30 million people around the world, and imposes substantial personal and societal burdens^1^. Typically, AD progresses through a preclinical phase with underlying biomarker abnormalities, then a prodromal state of mild cognitive impairment (MCI), and finally frank AD dementia^2^. Annually, 10–15% of patients diagnosed with MCI progress to AD dementia^3^. Identification of factors contributing to progression from MCI to AD is crucial for clinical prognostication and risk stratification to guide counseling and selection of potential treatments.

In the last decade, biomarkers from cerebrospinal fluid (CSF), positron emission tomography (PET), and magnetic resonance imaging (MRI) have been increasingly used in AD clinical and research studies to assess the degree of AD related pathology. Increased amyloid pathology measured by decreased CSF Aβ42 and increased cerebral amyloid on PET, as well as increased neuronal injury assessed by increased CSF tau, hypometabolism on FDG-PET, and atrophy on structural MRI, are important factors in assessing the degree of brain changes due to AD pathology and as a surrogate for prediction of progression in individuals with MCI^2^. In addition^4–6^, clinical measures such as Mini-Mental State Examination (MMSE) and Alzheimer’s Disease Assessment Scale-Cognition (ADAS-Cog) which reflect the current level of impairment in individuals have been shown to be useful for prediction of MCI progression^4–6^.

Two additional important factors in the context of MCI progression to AD are genetic factors and cognitive resilience. AD is a genetically complex disorder, with susceptibility thought to reflect the collective influences of multiple genetic risk and protective factors^7^. Other than the apolipoprotein E (*APOE*) ε4 allele, individual genetic variants associated with AD have shown modest population-level effect sizes, in keeping with current hypotheses about the genetic architecture of the disease^8^. Although the heritability of AD is thought to be 60–80%, beyond the well-studied effect of the *APOE* ε4 allele^9^, relatively little is known about the genetic factors specifically related to MCI-to-AD progression^10^, particularly regarding their added value above known biomarker profiles. Cognitive resilience represents the ability of an individual to delay the deleterious effects of neurodegenerative pathologies on onset of cognitive symptoms^11^. While cognitive resilience has been widely used to explain the pathology-cognition disconnect in cognitively unimpaired individuals with AD pathology (and normal cognition), its relative influence specifically on MCI-to-AD progression has not been fully evaluated^12^. Furthermore, the complex relationships among the well-known AD biomarkers, cognitive resilience, and genetic factors remain largely unknown and estimating the independent predictive values provided by each of these factors may open the door to alternative strategies to delay or prevent the onset of dementia.

Several machine learning (ML) based approaches have been proposed for predicting MCI-to-AD progression^4,5,13,14^ and clinical stage classification^15^ utilizing high-dimensional clinical and biomarker data among the potential predictors. A limitation of the ML-based approaches that utilize high-dimensional data is the potential for overfitting, such that the classifiers are so optimally trained to fit the primary dataset that they perform poorly on previously unseen test data, ultimately limiting the generalizability and wider interpretability^15^ of the predictive model. This is particularly important in this study because both a) an unbiased estimation of the contributions of cognitive resilience and genetic factors in predicting MCI-to-AD progression and b) the successful clinical translation of this technique, would require a generalizable model. However, prior studies on the predictability of MCI-to-AD progression have not performed satisfactory assessments of generalizability^4,5,13,14^. These approaches utilize non-linear classifications methods such as multi-kernel learning and artificial neural networks without establishing the need for non-linear models. Non-linear classification models are more susceptible to overfitting compared to linear classification models in problems involving high-dimensional data since they optimize relatively higher number of model parameters^16^. Whereas, a pitfall associated with linear models is that they provide suboptimal prediction performance when the data is not linearly separable. Therefore, our goals in this study with respect to the development of a predictive model are a) to understand whether linear classifiers are sufficient to provide clinically relevant accuracies in predicting MCI-to-AD progression based on a set of features derived from multiple modalities and b) to develop an analytical method that evaluates the generalizability of different classifiers and to aid model selection in clinical settings.

In this study, we used a machine learning-based approach and data from a well-characterized clinical cohort to identify individuals with MCI who rapidly progress to AD versus those with a more protracted course. Specifically, using a set of features derived from the ADNI multimodal biomarker and clinical dataset along with genetic factors and cognitive resilience measures, a linear model-based ML framework was developed for predicting MCI-to-AD progression. Based on a test of linear-separability^17^, we show that linear classification models perform just as good as non-linear models in this setting and a support vector machine classifier with a linear kernel provides the most generalizable performance with a test set area under ROC curve (AUC) value of 0.93. Using this framework, we evaluated the predictive values of previously underexplored factors of cognitive resilience and genetic factors and gauged their relative importance compared to commonly used CSF and imaging predictors. We found that, while the markers of AD pathophysiology (amyloid, tau, and neuronal injury) provided very high predictive values, the genetic factors and brain regions associated with cognitive resilience also displayed independent predictive values.

## Materials and Methods

### Study participants

This report utilized data from the Alzheimer’s Disease Neuroimaging Initiative (ADNI), a multisite longitudinal study of older adults representing clinical stages along the continuum from normal aging to AD^18^. All study participants provided written informed consent, and study protocols were approved by each local site’s institutional review board. Further information about ADNI, including full study protocols, complete inclusion and exclusion criteria, and data collection and availability can be found at http://www.adni-info.org/. All methods as stated on the website were performed with the relevant guidelines and regulations. Since all the analyses were performed on de-identified ADNI data which is publically available for download, IRB Review was not required. In addition, all methods were carried out in accordance with the approved guidelines.

In choosing ADNI participants to study, we used these inclusion criteria: i) the person had at least three years of follow-up; ii) the person had all the data modalities of interest (as specified below); and iii) the person was diagnosed with MCI at the baseline evaluation. We identified 135 participants who met those criteria. A total of 39 of the 135 progressed to AD within three years of the MCI diagnosis (and are referred to as the *MCI-P group*); the remaining 96 progressed to AD after three years or remained in MCI until their last follow-up after three years (and are referred to as the *MCI-NP group*).

### Predictive factors

**Figure 1:**
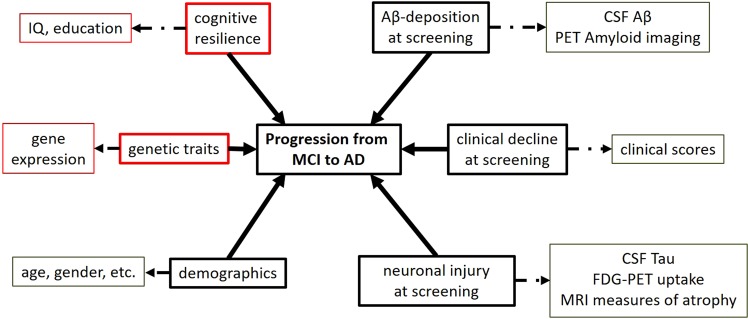
Factors that can predict progression from MCI to AD. The extent of Aβ-deposition, clinical decline, and neuronal injury at baseline represent the clinical severity of the disease in the MCI subjects. Cognitive resilience, genetic traits, and demographic factors are measures of heterogeneity within the cohort. Aβ-deposition is generally measured using CSF-Aβ and PET amyloid imaging. Neuronal injury is measured using CSF-Tau, FDG-PET, glucose uptake, and MRI atrophy measures. Clinical cognitive decline is measured via clinical scores such as Mini Mental State Examinations (MMSE). Cognitive resilience in a subject can be measured using IQ and level of education. The genetic traits of an individual can be measured using gene expression (RNA) measures. Demographic factors like age, gender, and disease risk factors can also influence the progression. Indicated using solid arrows are factors that influence MCI-to-AD progression and broken lines indicate measurements that were used to measure those factors. Factors and measurements highlighted in red are those that have not been studied in previous MCI-to-AD progression studies in conjunction with the rest.

Figure 1 illustrates potential factors that may impact progression from MCI to AD. Below we describe the specifics of the individual biomarkers utilized in this work.

All the biomarkers utilized in this work were downloaded from the LONI data archive (https://ida.loni.usc.edu/) where the pre-processed ADNI data are hosted.

#### Cerebrospinal fluid (CSF) biomarkers

CSF levels of amyloid beta (Aβ) and total (T-tau) and phosphorylated (P-tau) tau proteins were assayed by the ADNI Biomarker Core as previously described^19^.

#### Magnetic resonance imaging biomarkers

Structural MRI (SMRI) scans at baseline were downloaded and processed as described previously^20^. FreeSurfer v5.1 was used to obtain volume and thickness measures for standard regions of interest (ROIs) as surrogates for cerebral atrophy. We scaled the volumes by total intracranial volume. In addition, we also included volumetric measurements of hippocampal subfields.

#### Positron emission tomography (PET) biomarkers

Fluorodeoxyglucose (FDG) and F-florbetapir PET imaging from the baseline visit were analyzed as surrogates for neuronal injury and amyloid pathology, respectively^18^. For FDG-PET, AD-specific ROIs representing the temporal, angular, and posterior cingulate gyri were utilized^21^. For F-florbetapir PET, we included regional amyloid deposition assessed by standardized uptake value ratio (SUVR) in the temporal, parietal, and cingulate cortex, as well as a composite global measure of multiple regions^22^.

#### Cognition

Scores on the Mini-Mental State Examination (MMSE)^23^ at baseline were utilized as measures of cognitive performance.

#### Cognitive resilience

The number of errors on the American National Adult Reading Test (ANART) (which is an estimate of pre-morbid verbal IQ) and years of education were utilized as surrogate measures of cognitive resilience.

#### Genetic factors

Genotype and gene expression data from peripheral blood samples in ADNI were obtained as previously described^24^. In this study, we specifically analyzed *APOE* ε4 allele status (carrier vs. non-carrier) as well as expression data for the top genes with validated associations to AD (APOE, BIN1, CLU, ABCA7, CR1, PICALM, MS4A6A, CD33, and CD2AP) as listed in the AlzGene database^25^.

#### Demographics

Gender and age at the baseline visit were utilized in the predictive model.

#### Data aggregation and ML preprocessing

A total of 94 potential predictive factors were included for analysis. A matrix was generated with 135 rows (representing study participants) and 94 columns (representing the potential predictive factors for MCI-to-AD progression). Prior to further analysis, we centered and standardized all data on a feature-by-feature basis by subtracting the mean and then dividing by the standard deviation.

### ML-based prediction framework

**Figure 2:**
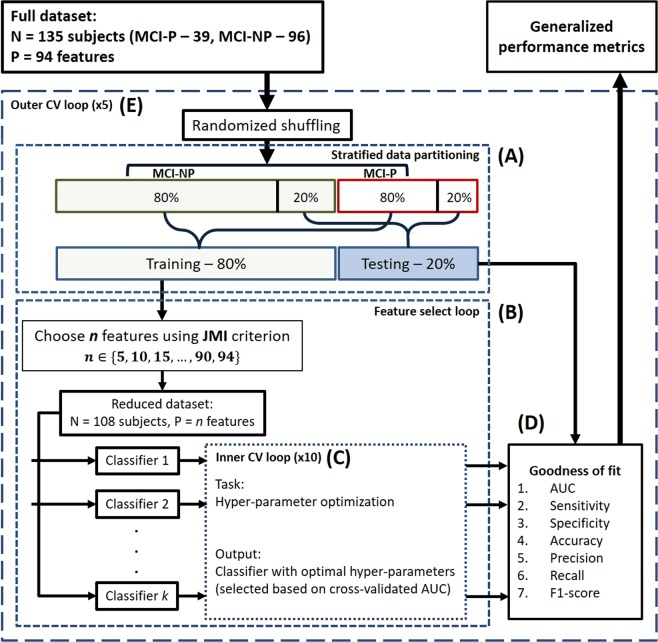
A flow diagram illustrating the prediction framework. The framework uses a machine learning-based approach to learn a classifier using 80% of the full dataset and to test its performance on the remaining 20% of the data. Specific details of each step in the framework are as follows. (**A**) Stratified data partitioning: After the order of the subjects is randomized, the MCI-NP and MCI-P groups are separately partitioned with an 80%-training/20%-testing split. The respective training and testing sets from MCI-NP and MCI-P groups are combined to form the overall training and testing data for a single cross-validation. (**B**) Feature select loop: The top n out of 94 features that best jointly correlate with the class labels (P-MCI or MCI-NP) are selected using the joint mutual information (JMI) criterion. (**C**) Inner CV loop: A combination of hyper-parameters is selected for each classifier based on a tenfold cross-validation. (**D**) Goodness-of-fit metrics: The classifier learned in the previous step is tested on the testing dataset and measured on its performance. (**E**) Outer CV loop: A fivefold cross-validation is utilized to produce generalized performance metrics accounting for non-uniformly distributed data.

Figure 2 shows a flow diagram of the prediction framework developed for this study. The framework consists of four major steps: information-theoretic feature selection, classifiers and hyper-parameter optimization, goodness-of-fit evaluation, and generalized performance evaluation. They are explained in the following paragraphs. A unique aspect of this workflow is the ability to specify the number of parameters optimized in the classification model by using an information-theoretic feature selection method. This is particularly useful in assessing the generalizability of a classifier as a function of the number of model parameters.

### Feature reduction using joint mutual information (JMI)

Mutual information between two random variables quantifies the amount of information shared between them. Mutual information is a more comprehensive measure of the relationships between random variables than statistical correlation-based approaches, which measure linear relationships only. Mathematically, mutual information is denoted by $I(Q;R)$ and defined for discrete random variables *Q* and *R* as shown in Eq. 1, where ${\bf{Q}}$ and ${\boldsymbol{ {\mathcal R} }}$ denote the alphabets of *Q* and *R*, respectively.

1 $$I(Q;R)={{\rm{\Sigma }}}_{q\in {\mathscr{Q}}}{{\rm{\Sigma }}}_{r\in  {\mathcal R} }p(q,r)log\frac{p(q,r)}{p(q)p(r)}$$

When *F*_*k*_ is one of the attributes in a set of attributes $\{{F}_{1},{F}_{2},\ldots {F}_{k}\}$ and *Y* is an outcome or class that can be predicted by the attributes, mutual-information-based approaches can be used to select the most predictive attributes. One such approach is to treat the attributes as independent random variables, rank them in descending order based on their mutual information with respect to the outcome *Y*, and select the top *n* number of attributes. One limitation of that approach is that useful and parsimonious sets of features should be both (i) individually relevant, and (ii) not highly correlated with each other. Joint mutual information shared between $\{{F}_{1},{F}_{2},\ldots {F}_{k}\}$ and *Y* is defined as shown in Eq. 2, where *F*_*k*_ and *Y* denote the alphabets of *F*_*k*_ and *Y*, respectively.

2 $$\begin{array}{ccc}I({F}_{1},{F}_{2},\ldots {F}_{k};Y) & = & {{\rm{\Sigma }}}_{{f}_{1}\in {{\rm{F}}}_{1}}{{\rm{\Sigma }}}_{{f}_{2}\in {{\rm{F}}}_{2}}\ldots {{\rm{\Sigma }}}_{{f}_{k}\in {{\rm{F}}}_{k}}{{\rm{\Sigma }}}_{y\in {\rm{Y}}}p({f}_{1},{f}_{2},\ldots {f}_{k},y)log\frac{p({f}_{1},{f}_{2},\ldots {f}_{k},y)}{p({f}_{1},{f}_{2},\ldots {f}_{k})p(y)}\end{array}$$

A JMI-based feature selection method starts with an empty set of attributes and iteratively adds *F*_*i*_s that, when added, provide the maximum increase in the joint mutual information shared between the set of attributes and the outcome^26^. It has been shown to be the most stable and flexible feature selection method^27^ among all the information-theoretic feature selection methods developed to date.

### Classification methods

We evaluated a number of classifiers in this study to understand the concepts of linear separability and generalizability in the context of predicting MCI-to-AD progression. Support vector machine (SVM), multiple kernel learning (MKL), and generalized linear models (GLM) with elastic-net regularization are the classifiers used in this study. SVM classifier, because it allows the transformation of features using linear and non-linear kernels, provides us the ability to evaluate the suitability of linear and non-linear classifiers for this problem. On the other hand, MKL allows the application of different kernel transformations for features from different modalities while optimizing more model-parameters than an SVM classifier. Although MKL and SVM are similar classification paradigms, MKL facilitates the integration of multiple modalities at the expense of potential overfitting. Finally, GLM classification with elastic-net regularization is an extension of the commonly known logistic regression classifier with additional regularization to minimize overfitting. In this study, we evaluated the overall generalizability of these classifiers that have distinct optimization objectives.

A support vector machine is a binary classifier that finds the maximum margin hyperplane that separates the two classes in the data^28^. Suppose that the data being classified are denoted by $X\in {{\mathbb{R}}}^{N\times P}$ (*N* subjects and *P* features), and that the data from subject *i* are denoted by ${X}^{(i)}\in {{\mathbb{R}}}^{P}$. Let us also use $Y\in {\{-1,1\}}^{N}$ to denote the class labels for all the subjects (where $-1$ and $1$ are numerical labels for the two classes), and ${Y}^{(i)}\in \{-1,1\}$ to denote the class label for subject *i*. The optimization problem to find the optimal hyperplane (described by weights $W\in {{\mathbb{R}}}^{P}$ and intercept term $b\in {\mathbb{R}}$) is shown in Eq. 3.

3 $$\begin{array}{c}{{\rm{\min }}}_{{\rm{w}},{\rm{b}}}\frac{1}{2}\parallel W{\parallel }^{2}\\ {\rm{Subject}}\,{\rm{to}}\,{Y}^{(i)}({W}^{T}{X}^{(i)}+b)\ge 1,\,i\in \{1,\ldots ,N\}\end{array}$$

Once the optimal hyperplane $[{W}_{opt},{b}_{opt}]$ has been found, the predicted class label for subject *i* is obtained as the sign of ${W}_{opt}^{T}{X}^{(i)}+{b}_{opt}$. This formulation assumes that the data are fully linearly separable between the two classes. When that is not the case (but there is still a linear classification task), slack variables and a tolerance parameter (box-constraint) can be introduced to obtain separating hyperplanes that tolerate small misclassification errors.

Dual formulation of SVM has received considerable interest due to its ability to use different kernel transformations of the original feature space without altering the optimization task, and due to its advantages in complexity when the data are high-dimensional^29,30^. The dual form of the above optimization problem can be written as shown in Eq. 4, where the operation $\langle .\rangle$ denotes an inner product between two vectors.

4 $$\begin{array}{c}{\max }_{{\rm{\alpha }}}{{\rm{\Sigma }}}_{i}{\alpha }_{i}-\frac{1}{2}{{\rm{\Sigma }}}_{i,j}\,{\alpha }_{i}\,{\alpha }_{j}{Y}^{(i)}{Y}^{(j)}\langle {X}^{(i)},\,{X}^{(j)}\rangle \\ {\rm{subject}}\,{\rm{to}}\,{{\rm{\Sigma }}}_{i}{\alpha }_{i}{Y}^{(i)}=0\,and\,{\alpha }_{i}\ge 0\,{\alpha }_{i}\ge 0\,i\in \{1,\ldots ,N\}\end{array}$$

Notably, the dual formulation can be expressed simply in terms of the inner product between ${X}^{(i)}$ and ${X}^{(j)}\,$(which is a scalar value). Therefore, any transformation of the features can replace the original features, i.e., $\langle {X}^{(i)},\,{X}^{(j)}\rangle$ can be replaced by $\langle \varphi ({X}^{(i)}),\,\varphi ({X}^{(j)})\rangle$, where $\varphi$ is a feature mapping. Furthermore, for any such feature transformation $\varphi$, we can define a kernel function *K* such that $\langle \varphi ({X}^{(i)}),\,\varphi ({X}^{(j)})\rangle =K({X}^{(i)},\,{X}^{(j)})$. With the kernel transformation, the cases where the original data are not linearly separable, may be solved as transforming the data to higher dimensions may introduce linear separation in the transformed domain. Linear, radial basis function (RBF), and polynomial kernels are widely used kernels in this context.

Multiple kernel learning is a classification technique that builds upon the above property of SVM and introduces additional variables in order to weight the kernel transformation of^31^ individual features. It does so by replacing the kernel function $K({X}^{(i)},{X}^{(j)})$ with $\sum _{m=1}^{P}{\beta }_{m}{K}_{m}({X}^{(i)}(m),{X}^{(j)}(m))$, where ${K}_{m}\,$is the kernel function of the *m*^th^ feature and ${\beta }_{m}$ is its weight. This technique is particularly useful when the data contain different modalities of features that need to be weighted differently. In a standard kernel-SVM, either the kernel transformation is uniformly weighted, or the weights are determined manually. It has been shown that the same dual formulation of SVM can be utilized to find the optimal weightings of the kernel transformation under the constraint that ${{\rm{\Sigma }}}_{m=1}^{{\rm{P}}}\,{\beta }_{m}=1$.

Logistic regression is a subclass of generalized linear models (GLM) and is well-suited for binary classification tasks^32^. It models the response variable as a binomially distributed random variable whose parameters are described by predictor variables and model parameters. Regularization of the model parameters is utilized to avoid effects related to overfitting. The objective function of a regularized GLM model is

5 $${\min }_{{\rm{\beta }},{{\rm{\beta }}}_{0}}\frac{1}{N}{{\rm{\Sigma }}}_{I=1}^{{\rm{N}}}[{Y}^{(i)}\,\mathrm{log}\,h({X}^{(i)})+(1-{Y}^{(i)})\mathrm{log}(1-h({X}^{(i)}))]+\lambda {P}_{\alpha }(\beta )$$

where $h(x)$ is the logistic function defined as $\frac{1}{1+{e}^{-x}}$ and ${P}_{\alpha }(\beta )$ is the regularization term with the elasticity parameter $\alpha$. The regularization term in general might contain both ${L}_{1}$-norm terms and ${L}_{2}$-norm terms, a situation that is commonly referred to as *elastic net regularization* with ${P}_{\alpha }(\beta )=\frac{1-\alpha }{2}{\Vert \beta \Vert }_{2}^{2}+\alpha {\Vert \beta \Vert }_{1}$^33^. The elasticity parameter $\alpha$ ranges in $(0,1]$ with values of *α* → 0 approaching ridge regression and *α* → 1 approaching LASSO regression.

### Hyper-parameter optimization

The hyper-parameters of the three classifiers are SVM’s kernel, kernel scale σ, and box-constraint $C$, MKL’s kernel, and GLM’s penalty term $\lambda$ and elastic-net parameter $\alpha$. The optimal values of hyper-parameters are chosen by performing a grid-search of parameter values with a k-fold cross-validation within the training dataset.

### Goodness-of-fit evaluation and generalized performance metrics

Goodness of fit of a classifier is evaluated by i) predicting the classes of the test dataset by using the classifier that was trained on the training dataset, and ii) comparing the predictions against the true class labels of the test dataset. The comparison is performed using standard performance metrics such as receiver operating characteristics (ROC) curve analysis, area under ROC curve (AUC), sensitivity, specificity, accuracy, precision, recall, and F1-score.

Because of heterogeneity in the data, choosing one partition of training and test datasets is not sufficient to credibly evaluate the performance of a classifier. A common practice to obviate the effect of heterogeneity in the data is to perform an k-fold training-testing cross-validation of the dataset. One run of this procedure is carried out by randomly choosing a subset of the dataset as training data, and testing on the rest of the dataset. That is repeated $n$ different times, and the performance metrics of all $n$ evaluations are averaged. In addition to this, the proportion of the different classes in the randomly chosen training dataset was kept constant in our approach via a stratified data-partitioning to eliminate any variability in the performance of the classifiers due to class imbalances in the data^16^. We performed those extra steps to obtain a generalizable set of performance metrics for the analyzed classifier.

### Implementation of the prediction framework

The prediction framework was implemented in MATLAB version R2017b and the code is publicly made available at https://gitlab.engr.illinois.edu/varatha2/adni_mci2ad_prediction. We utilized a standard fivefold cross-validation with 80% training data and 20% testing data to evaluate the performance of the classifiers. Training and testing data selection was performed using the stratified data-partitioning approach explained previously. The JMI criterion was used to identify $n\in \{5,10,\ldots ,\,90,\,94\}\,$features based on the training dataset and corresponding labels. Using the reduced dataset, we trained SVM with a linear kernel, SVM with an RBF kernel, MKL with a linear kernel, LR with elastic net regularization, and RF. The highest average AUC obtained with a tenfold cross-validation within the training set was used as the selection criterion for the optimal hyper-parameters. We generated performance metrics for each classifier with the best identified hyper-parameters by predicting the labels of the test set and comparing them against the true class labels of the test set. The performance metrics of all five of the (outer) cross-validation runs were averaged to generate generalized performance metrics.

### Test for linear separability

Linear separability of a dataset with two classes signifies that a linear classifier (or a hyperplane) can separate the two classes in the data as well as nonlinear classifiers can. We show that by using a slight modification of the *histogram of projections*^17^ method, which is considered a test for class separability using SVM classification. The histogram of projections method^17^, is as follows. An SVM classifier is trained using the training data and a chosen kernel. Then, the test samples are projected on the 1-dimensional line perpendicular to the maximum margin hyperplane learned by the SVM model. Histograms of these projections for the two different classes are compared to evaluate the degree of separation achieved by the classifier. In our approach, we applied a sigmoid transformation to the projections of the test samples to obtain likelihood probabilities, and then, based on the likelihood probabilities, we created histograms of the two classes. This modification was performed in order to eliminate the effects of scaling when different kernel transformations are applied in SVM classification and to maintain the range of histograms within a closed set $[0,1]$.

## Results

Our results are organized as follows. Table 1 represents the overall summary statistics of the dataset analyzed in this study with some important demographic factors. First, we present the results of our evaluation of whether linear models are sufficient to classify this dataset. Second, we report the analytic method we developed to evaluate generalizability and the results on the generalizability of the analyzed linear classifiers. Third, we report the relative importance of the biomarkers and clinical variables utilized in this study evaluated using the most generalizable classifier selected based on the previous result. Finally, we report the analysis based on LASSO regression^34^ and a set of identified features that provide independent information in predicting MCI-to-AD progression.

**Table 1:** Summary characteristics of participants at baseline.

	MCI-P (n = 39)	MCI-NP (n = 96)
Average age (and range of ages)	72 (55–84)	71 (55–88)
Number of females (and percent female)	19 (49%)	48 (50%)
Average years of education (and range)	16 (9–20)	16 (12–20)
Number of *APOE* carriers (and percent)	6 (15%)	3 (3%)

Here we list the summary statistics of the participants in this study.

### Linear separability

First, we evaluated the linear separability of the data in order to understand whether linear classifiers, which are less susceptible to overfitting, were sufficient to perform prediction of MCI-to-AD progression. We applied the modified histogram of projections method to our dataset by training two SVM classifiers with linear and RBF kernels and by plotting histograms of sigmoid transformations of the projections of test samples.

**Figure 3:**
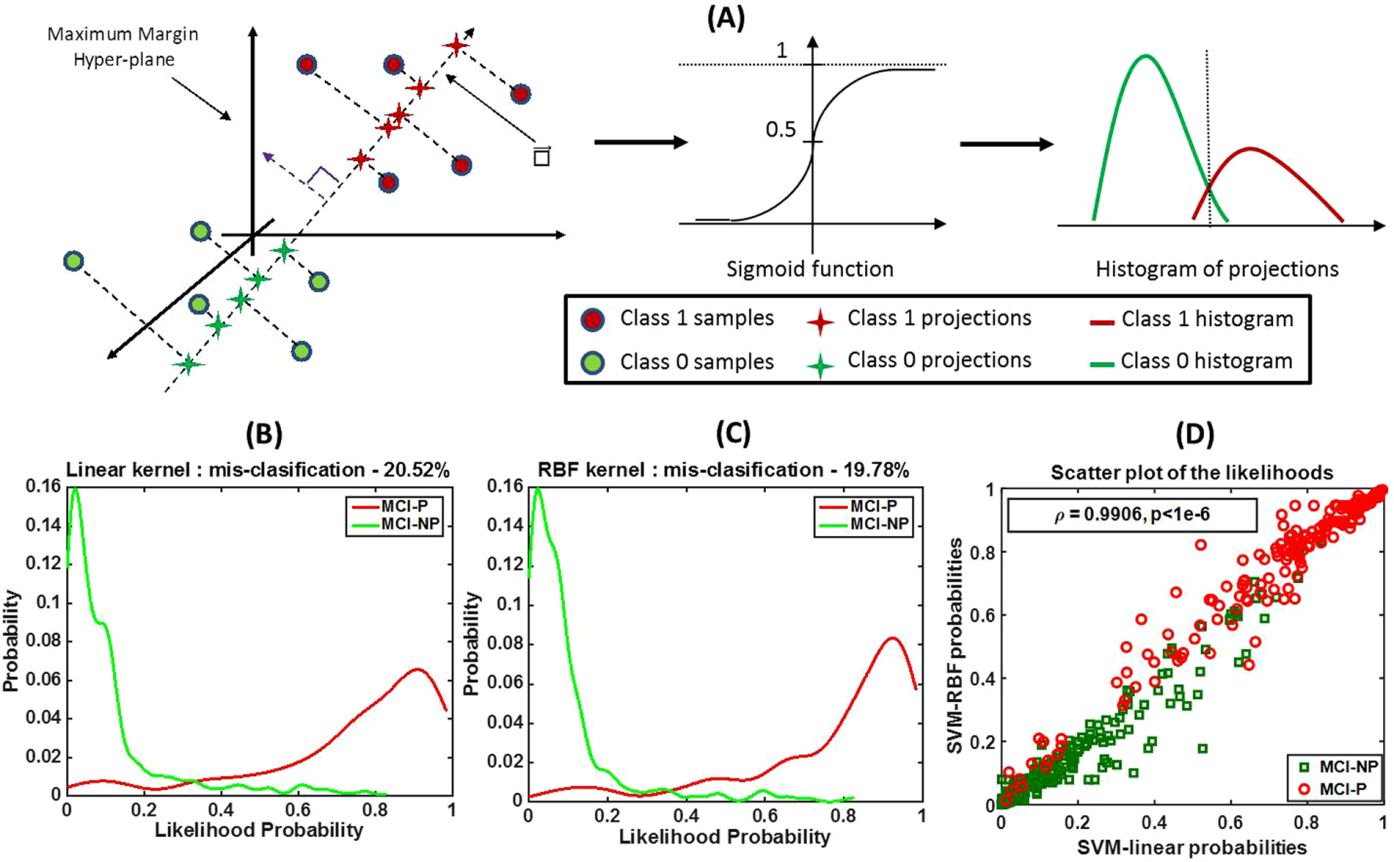
An illustration of the method that evaluates linear separability of the data. We utilize a slightly modified version of the histogram of projections method to evaluate linear separability of the data. (**A**) A maximum margin hyperplane is learned using SVM with a choice of kernel. All samples are projected onto the line perpendicular to the hyperplane to obtain the projections. The projection lengths are transformed to a probability value via the sigmoid function. Histograms of the probabilities for the two classes are plotted separately. (**B**) Histograms of the probabilities of the MCI-P and MCI-NP samples in our dataset obtained using a linear kernel. (**C**) Histograms of the probabilities of the MCI-P and MCI-NP samples in our dataset obtained using an RBF kernel. (**D**) A grouped scatter plot of the probabilities obtained using linear and RBF kernels for MCI-P and MCI-NP classes. The similar histogram shapes and similar misclassification errors in (**B**,**C**), and the high correlation (${\boldsymbol{\rho }}$ = 0.99, p < 1e-6) between the probabilities obtained using the two kernels, indicate that linear and nonlinear kernels result in similar boundaries for classification; hence, this dataset is linearly separable.

Figure 3A shows a generic illustration of our approach based on the histogram of projections method. Figure 3B,C show the histograms obtained using our approach for linear and RBF kernels, respectively. The two histograms have similar shapes, and the misclassification errors obtained by choosing an appropriate threshold are also close (20.52% for the linear kernel and 19.78% for the RBF kernel). Figure 3D shows a grouped scatter plot of the probabilities obtained using linear versus RBF kernels for the MCI-P and MCI-NP classes. There is a significantly high correlation between the probabilities obtained using the two kernels ($\rho$ = 0.99, p < 1e-6), indicating that the classification boundaries learned using the linear and nonlinear kernels are similar. This experiment suggests that the dataset being analyzed is linearly separable. Hence, we limited our analyses to linear classification techniques for the rest of this paper.

### Generalizability of classifiers

**Figure 4:**
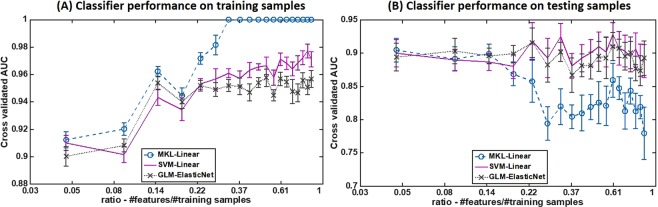
An evaluation of generalizability of linear classifiers. Three linear classifiers—multiple kernel learning (MKL) with linear kernels, support vector machine (SVM) with a linear kernel, and generalized linear model (GLM) with elastic-net regularization—were trained multiple times using 80% of the data as the training set but with a variable number of features each time. We plotted the cross-validated AUCs with their standard deviations against the ratio $\frac{{\rm{number}}\,{\rm{of}}\,{\rm{features}}\,{\rm{used}}\,{\rm{in}}\,{\rm{training}}}{{\rm{number}}\,{\rm{of}}\,{\rm{training}}\,{\rm{samples}}}$ for both training (**A**) and testing (**B**) sets. While all the classifiers show an increasing AUC trend on the training set with increased numbers of features used in training, only SVM and GLM show a relatively steady trend on the test set. MKL on the other hand, shows a decreasing testing AUC trend with increased numbers of features used in training.

Second, we evaluated the overfitting characteristics of the linear classifiers in order to identify models that were likely to provide highly generalizable performance on this dataset. Three linear classifiers—multiple kernel learning (MKL) with linear kernels, support vector machine (SVM) with linear kernel, and generalized linear model (GLM) with elastic-net regularization—were trained using 80% of the whole dataset as the training set but using only a subset of all the features. We selected the subset (out of a total of 94 features) using the joint mutual information (JMI) criterion, by varying the number of features used each time as {5, 10, 15, …, 85, 90, 94}. We utilized tenfold stratified cross-validation to obtain average training and testing area under ROC curve (AUC) metrics and their respective standard deviations on each occasion. Figure 4 shows plots of the cross-validated AUCs with their standard deviations against the ratio $\frac{{\rm{number}}\,{\rm{of}}\,{\rm{features}}\,{\rm{used}}\,{\rm{in}}\,{\rm{training}}}{{\rm{number}}\,{\rm{of}}\,{\rm{training}}\,{\rm{samples}}}$ for both training (4A) and test (4B) sets. On the training set, the AUCs of all the classifiers showed better performance with increasing numbers of features used in training. But on the test set, only SVM and GLM showed a relatively steady trend in the AUC. MKL, on the other hand, showed a decreasing testing AUC trend with increasing numbers of features used in training. Those data suggest that the MKL classifier may be overfitting the training data when the ratio $\frac{{\rm{number}}\,{\rm{of}}\,{\rm{features}}\,{\rm{used}}\,{\rm{in}}\,{\rm{training}}}{{\rm{number}}\,{\rm{of}}\,{\rm{training}}\,{\rm{samples}}}$ is not small (it is >0.14 in this case).

Those results suggest that both SVM with a linear kernel and GLM with elastic-net regularization have good generalizability (with a consistent AUC value of approximately 0.9) regardless of the ratio of the number of features to the number of training samples, and MKL had good generalizable performance only when this ratio was small. Further, particularly for this dataset, all linear classifiers showed a reasonable test set predictability when the number of features was appropriately chosen during the training phase.

### Prediction performance of linear classifiers

Based on the previous experiment, we selected the subset of features that provided the most generalizable predictive performance for each linear classifier. Table 2 lists all the cross-validated goodness-of-fit metrics (AUC, sensitivity, specificity, accuracy, precision, recall, and F1-score) obtained using MKL with linear kernels, SVM with a linear kernel and GLM with elastic-net regularization, with their respective subsets of features. All linear classifiers produced comparable results, while SVM with a linear kernel provided the best result.

**Table 2:** Cross-validated goodness-of-fit metrics for linear classifiers.

	AUC	Sensitivity	Specificity	Accuracy	Precision	Recall	F1-score	Training size	#Features
MKL-Linear	0.90(0.02)	0.86(0.03)	0.78(0.03)	0.80(0.02)	0.63(0.03)	0.86(0.03)	0.72(0.03)	80%	5
SVM-Linear	0.93(0.02)	0.93(0.03)	0.77(0.03)	0.81(0.02)	0.64(0.03)	0.93(0.02)	0.75(0.03)	80%	65
GLM-Elastic Net	0.92(0.02)	0.91(0.02)	0.76(0.02)	0.81(0.02)	0.62(0.03)	0.91(0.04)	0.74(0.03)	80%	25

Here we list the goodness-of-fit metrics (AUC, sensitivity, specificity, accuracy, precision, recall, and F1-score) obtained for the test dataset (20% of the whole dataset), using the subset of features that provided the most generalizable result, as shown in Fig. 4. Their average values and standard deviations were computed using a tenfold stratified cross-validation.

### Predictive ability of individual modalities

**Figure 5:**
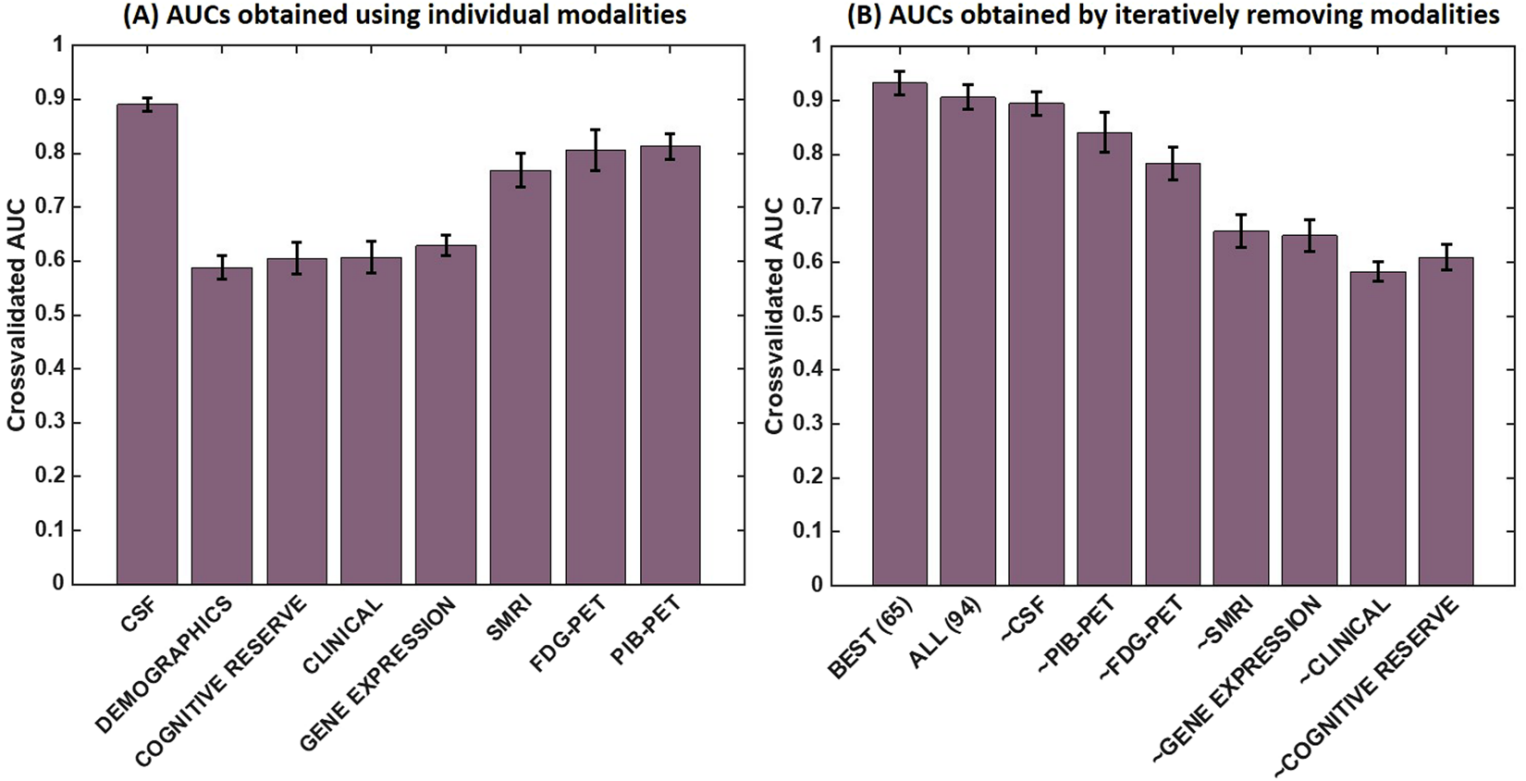
An evaluation of the relative predictive abilities of modalities. (**A**) The cross-validated AUCs obtained using an SVM classifier with a linear kernel separately for each of the modalities. (**B**) The AUCs obtained by iteratively removing the modalities in a descending order based on AUCs obtained in (**A**). (Modalities with high AUC values per (**A**) were removed first.) In (**B**), “~X” indicates that modality X was removed while the modalities that are less predictive than X were kept, to obtain the respective AUC.

To understand the predictive ability of individual modalities in a statistically impartial manner, we restricted our analysis in this subsection to only one linear classifier. Based on Table 2, we chose the SVM classifier with a linear kernel, as it provided the best performance in terms of the AUC metric. Then, we repeated our classification procedure using a stratified tenfold cross-validation for each of the modalities (using all the variables in the respective modality each time). Figure 5A shows a bar-chart of the average AUCs obtained by individual modalities. CSF proteomic markers (including Aβ, total-Tau, and phosphorylated-Tau) provided the best individual prediction capability, followed by the imaging markers in the order amyloid PET, FDG-PET, and SMRI. Gene expression, clinical, cognitive resilience, and demographic markers showed lower predictive ability than did CSF and imaging-based markers. Figure 5B shows a different bar-chart of the average AUCs obtained by iteratively removing the respective features of the individual modalities in a descending order based on their individual predictive abilities seen in Fig. 5A. A gradual decline in the AUC is observed, with the largest drop occurring when SMRI features were removed.

### Best predictors of MCI-to-AD progression

Next, we sought to identify a minimal set of predictors required to model MCI-to-AD progression. This was motivated by an observation about Fig. 5: although CSF features provided the best individual AUC (Fig. 5A), the removal of CSF features from the data (Fig. 5B) did not result in a substantial drop in the AUC (the reduction in AUC is within the error limits). That suggests that other modalities provide overlapping information that may be correlated with the CSF markers, and also indicates that all the modalities might have a notable level of correlation with each other. We used a generalized linear model (GLM) classification method with L1 regularization (known as *LASSO regression*) to identify a sparse set of features with minimal within-correlation and maximal prediction potential of clinical progression^34^. We chose to use LASSO regression instead of SVM classification for this task because the feature weights assigned by the SVM classifier with a linear kernel do not represent the independent predictive values of individual features when the features themselves are correlated with each other. However, the L1-norm-based regularization of LASSO regression may enable identification of the smallest possible set of predictors, since it penalizes the classifiers that utilize a large number of features in the resulting predictive function. Table 3 shows the results from this approach, identifying the factors that provided the best spread of independent information to predict MCI-to-AD progression. This model included PET, MRI, and CSF variables in addition to age and expression of *CR1* (complement receptor 1) and was able to predict MCI-to-AD progression with an AUC of 0.92.

**Table 3:** A minimal set of markers that are predictive of MCI-to-AD progression. A generalized linear model (GLM) classifier with L1 regularization was utilized to identify a small set of minimally correlated features that can predict MCI-to-AD progression.

CSF	PIB-PET	FDG-PET	SMRI	Genetic	Demographics
Total-Tau	COMPOSITE REFNORM	Angular-Left	Cortical Thickness Average of Insula	CR1	AGE
Amyloid Beta	TEMPORAL	Temporal-Left	Cortical Thickness Average of SuperiorFrontal		
Phosphorylated-Tau		Cingulum-Bilateral	Cortical Thickness Average of InferiorParietal		
		Temporal-Right	Cortical Thickness Average of Parahippocampal		
			Cortical Thickness Average of MedialOrbitofrontal		
			Cortical Thickness Average of CaudalAnteriorCingulate		
			Cortical Thickness Average of SuperiorParietal		
			Cortical Thickness Average of IsthmusCingulate		
			Cortical Thickness Average of ParsTriangularis		
			Cortical Thickness Average of PosteriorCingulate		
			Hippocampal Subfield Volume of Subiculum		

Those features are categorized here based on their feature modality.

## Discussion

In this work, we developed an accurate and generalizable machine learning-based methodology for predicting short-term progression of MCI to AD dementia in a well-characterized clinical cohort. A unique aspect of this work was our evaluation of important genetic markers and cognitive resilience markers in addition to the neuroimaging biomarkers and clinical examination. The results suggest that a combination of selected neuroimaging, blood and CSF biomarkers, and demographic traits reflect the underlying pathophysiology and factors that drive clinical progression in the AD spectrum. The methodology allowed us to discover that expression of *CR1* and AD-pattern neuropathology and neurodegeneration (MRI measures in the frontal lobes) in brain regions associated with cognitive resilience added independent predictive values in predicting MCI-to-AD progression. We also evaluated the relative importance of individual predictors and identified a minimal set of predictors that are important for predictive modeling of MCI progression to AD.

### Predictors of MCI progression to AD

The broad pathogenesis of AD has been well-described conceptually, from initial alterations in molecular and cellular pathology to neurodegeneration and eventually to clinical impairment sufficient to cause dementia^2^. However, it is not yet fully understood what specific factors “shift the curve” to either promote or inhibit the development of dementia. As a result, we initially approached our study in a relatively unbiased fashion, casting a broad net for potential predictive factors out of multidimensional clinical, neuroimaging, and other biomarker data. Through our ML approach, we found that measures of AD neuropathology (CSF amyloid and tau and cerebral amyloid assessed by PET) and neuronal injury (assessed by FDG-PET and structural MRI) explained the most variance in separating fast versus slow progression from MCI to AD dementia. The importance of biomarkers in predicting progression has been studied in the past, but the approach we took allowed us to rank various predictors (including the biomarkers) as well as identify the minimal predictor set that are key to the overall models. When the biomarkers were excluded, predictors such as cognitive performance, cognitive resilience, and expression of selected genes individually explained less of the outcome variance and collectively appeared to offer less new information to the predictive model. Our results support the hypothesis that CSF biomarkers and imaging can be used as surrogates for neuropathology and brain health and can serve as key indicators of future MCI prognosis.

### Information added by genetic factors and cognitive resilience

An important aspect of this work is the incorporation of genetic factors and cognitive resilience into our predictive model. Most cases of AD are thought to be genetically complex, with multiple factors presumed to contribute to susceptibility and protection, with the largest known factor being the *APOE* ε4 allele^35^. Understanding of the genetic architecture of AD has greatly expanded over the last decade as a result of genome-wide and rare-variant association studies, among other approaches^7^. However, the specific genetic factors that influence progression at various clinical stages of AD are still not well-characterized. Our ML approach identified expression of *CR1* as a key factor in predicting fast versus slow progression to AD dementia, which is a novel finding of this study. Interestingly, our final minimal set model included *CR1* expression but did not include *APOE* ε4 allele status, suggesting that the former provided unique information for clinical course prediction while the latter was already represented by surrogate biomarkers, specifically amyloid deposition^36^. Polymorphisms (genotype variations) in *CR1* have been associated with AD status and endophenotypes in numerous large-scale studies^37–42^. *CR1* encodes a receptor involved in complement activation, a major immune mechanism with a wide array of functions; it has been proposed that it impacts the clearance of amyloid in AD^43^. Previous findings on *CR1* have been illuminated by a more recent and extensive body of work highlighting immune system pathways as potential cruxes in AD pathophysiology^44–48^. Our new findings relating *CR1* expression to progression from MCI to AD dementia provide further validation of that previous work and argue for greater focus on *CR1—*and on genetic variation in MCI-to-AD progression more broadly^49^—to enable better understanding of the mechanisms underlying AD and its clinical trajectories.

It is not unexpected that in our ML-based prediction model for MCI to AD progression, *CR1* gene expression contributed relatively less to explaining variance than biomarkers of neuropathology and neuronal injury. Surrogates of pathology (fluid biomarker and neuroimaging) reflect the extent of disease related changes and are likely more proximal to clinical manifestations of disease compared to gene expression which may be upstream of these changes and thus possibly modifiable by concomitant forces over time. An example is the relationship between *APOE* ɛ4 and amyloid: while *APOE* ɛ4 is a key driver of amyloidosis, the measured effect of amyloid load on cognition is significantly stronger than the impact of *APOE* ɛ4 on cognition^36,50^. Complementary methods to incorporate collective effects of multiple genes, including pathway analyses and polygenic risk scoring^51–54^, could be incorporated into our ML approach in future. In addition, for this study we analyzed microarray gene expression (rather than genotype) data, which offers the conceptual advantage of being a dynamic (rather than static) marker of late-life conditions but which has the disadvantage of representing a downstream effect of influences earlier in life with the potential to be modified by interactions with other heritable and non-heritable factors. Finally, for this study we limited the focus to the AlzGene Top 10 list, but other genes with less well-known population-level effects on case-control status may have larger contributions to late-life clinical progression which could be discovered with an unbiased genome-wide approach. Despite these constraints, our approach serves as a proof of concept that incorporating genetic data can add value to ML-based clinical prediction models and highlights *CR1* for further study on its potential effects at the inflection of MCI to AD progression.

Although cognitive resilience has been shown to contribute significantly to delaying the onset of clinical impairment, neither education nor verbal IQ was identified as a key predictor of MCI progression. There are two possible explanations for those results. (i) Preservation of structure or lack of atrophy, as observed via MRI, may be a better surrogate of resilience than are estimates of pre-morbid IQ or educational attainment. It has been shown that cognitive resilience is captured well by greater volume and metabolism, especially in the frontal and cingulate regions^55^. (ii) Cognitive resilience may be more relevant before the onset of cognitive symptoms or impairment^12^, and thus may be of less importance in cognitively impaired individuals, who are the focus of this study.

### Strengths of the computational approach

We presented a machine learning-based prediction framework for predicting MCI-to-AD progression using state-of-the-art classification techniques under a widely accepted cross-validation setup that accounts for sample bias and unbiased hyper-parameter optimization. Our results indicate that linear classifiers are sufficient to identify patients who have the potential to progress from MCI to AD within 3 years, via the use of multimodal measurements including imaging, CSF, genetic, and clinical data. The reason for this might be that all the features were engineered prior to application of machine learning in such a way that a dichotomy within a feature correlates with disease progression. Linear classifiers are less susceptible to overfitting and thus can be appropriate for clinical translation. In addition, we developed an analytic method for evaluating the generalizability of three different linear classification approaches, namely GLM-ElasticNet, multi-kernel SVM with linear kernels, and standard SVM with a linear kernel. Multi-kernel SVM showed a slightly greater tendency to overfit than the other linear classifiers in our experiments. Because multi-kernel SVM optimizes twice as many model parameters as standard SVM or GLM, it seems that the number of samples used in this study was not enough to train multi-kernel SVM in a generalizable manner. However, as indicated in the experiments, when the number of features used in the classification was small, multi-kernel SVM seemed to provide a more generalizable performance compared to when more features were used in the classification, since there were fewer optimized model parameters in the former scenario. Even so, standard SVM and GLM provided better generalizability and achieved higher classification accuracies with more features. In comparison with previously published predictors of MCI-to-AD progression, our approach achieves superior predictive performance, with a 0.93 area under ROC curve providing a 6% improvement over the current best predictor^4^, which utilized only CSF and imaging modalities for the prediction of short-term MCI-to-AD progression. It is our firm belief that AUCs of test sets are the most reliable measure of a classifier’s general performance, and hence that our evaluation presents a fair assessment of the proposed ML-based predictor.

### Limitations and future directions

Our study has several limitations, which may be addressed by future work. We used publicly available data from the ADNI and ultimately analyzed only a modest sample, because we could only use data from patients for whom we had complete data for all the assessed predictive variables. Replication of our methods in similar and larger cohorts would help validate our approach. In addition, application of our final model to an independent dataset could extend our findings by assessing their success in predicting clinical progression from known baseline data. Further, use of alternative surrogate variables—such as verbal episodic memory performance (rather than MMSE scores) as a reflection of cognitive impairment, and lifetime intellectual enrichment (rather than, or in addition to, the AMNART or educational attainment) as a reflection of cognitive resilience—as well as newer biomarkers such as Tau-PET might produce a more optimal model. We also chose to focus on differentiation of faster versus slower progression from MCI. However, it is not yet known whether the factors that are important at that clinical stage are more broadly generalizable to earlier stages of AD at which clinical intervention might have greater impact. Finally, a broader investigation of genetic factors, including genome-wide genotype and expression data, might increase the accuracy of our model and raise additional loci for follow-up.

In conclusion, we used a machine learning approach to integrate data from multiple sources to predict progression from MCI to AD dementia. By analyzing the linear-separability of the dataset, we established that linear classifiers are sufficient for predicting MCI-to-AD progression in the proposed setting. Furthermore, we also developed an analytical method to assess the generalizability of classifiers based on the number of model parameters. We also showed that, the markers of AD pathophysiology (amyloid, tau, and neuronal injury) provided very high predictive values, the genetic factors and brain regions associated with cognitive resilience also displayed independent predictive values. Our findings, particularly in relation to the impact of genetic factors and cognitive resilience in the setting of biomarkers and clinical tests, warrant further investigation using larger datasets and may have downstream benefits leading to improved prognostication and risk stratification in AD.

## Data Availability

Data used in preparation of this article were obtained from the Alzheimer’s disease Neuroimaging Initiative (ADNI) database (http://adni.loni.usc.edu). Thus, the investigators within the ADNI contributed to the design and implementation of ADNI and/or provided data, but did not participate in this analysis or the writing of this report. A complete listing of ADNI investigators can be found at http://adni.loni.usc.edu/wpcontent/uploads/how_to_apply/ADNI_Acknowledgement_List.pdf/. For additional details and up-to-date information, see http://www.adni-info.org.
